# Prevalence of Diabetes among Migrant Women and Duration of Residence in the United Arab Emirates: A Cross Sectional Study

**DOI:** 10.1371/journal.pone.0169949

**Published:** 2017-01-18

**Authors:** Syed M. Shah, Raghib Ali, Tom Loney, Faisal Aziz, Iffat ElBarazi, Salma Al Dhaheri, M. Hamed Farooqi, Iain Blair

**Affiliations:** 1 Institute of Public Health, College of Medicine and Health Sciences, United Arab Emirates University, Al Ain, United Arab Emirates; 2 Public Health Research Center, New York University Abu Dhabi, Abu Dhabi, United Arab Emirates; 3 Ambulatory Health Services, SEHA, Al Ain, United Arab Emirates; 4 Dubai Diabetes Center, Dubai Health Authority, Dubai, United Arab Emirates; Western Sydney University, AUSTRALIA

## Abstract

**Background:**

The prevalence rate of type 2 diabetes mellitus (T2DM) is one of the highest in United Arab Emirates (UAE), however data for the expatriate population is limited. This study aimed to identify the prevalence of T2DM amongst migrant women and test the hypothesis that acculturation (measured by years of residency) is associated with an increased risk of T2DM.

**Methods:**

This was a cross-sectional study and we recruited a representative sample (n = 599, 75% participation rate) of migrant women aged 18 years and over in Al Ain, UAE. The American Diabetes Association criteria were used to diagnose T2DM. An adapted WHO STEPS questionnaire was used to collect socio-demographic, lifestyle and clinical data. Logistic regression analysis was performed to identify correlates of T2DM including length of UAE residence.

**Results:**

The mean age of participants was 34.1 (± 9.5) years. Of the study participants, based on HbA1C levels, 18.6% (95% CI: 13.9–24.4) had prediabetes and 10.7% (95% CI: 7.2–15.6) had T2DM. Prevalence of prediabetes was 8.5% for Filipinos, 16.7% for Arabs and 30.3% for South Asians. Similarly the prevalence of T2DM was 1.7% for Filipinos, 12.2% for Arabs and 16.7% for South Asians. Significant correlates of overall T2DM (measured and known diabetes) included length of UAE residence for more than 10 years (Adjusted Odds Ratio [AOR] 2.74, 95% CI: 1.21–6.20), age ≥40 years (AOR = 3.48, 95% CI: 1.53–7.87) and South Asian nationality (AOR 2.10, 95% CI: 0.94–4.70).

**Conclusion:**

Diabetes is a significant public health problem among migrant women in the UAE, particularly for South Asians. Longer length of residence in the UAE is associated with a higher prevalence of diabetes.

## Introduction

Type 2 diabetes mellitus (T2DM) is one of the major causes of premature morbidity and mortality worldwide with the World Health Organization (WHO) reporting that one in ten adults worldwide had T2DM in 2014 [1]. Current projections estimate that globally T2DM will be the seventh leading cause of death in 2030 [2]. Previously, the United Arab Emirates (UAE) reported the second highest prevalence of T2DM (18.7%) in the world, among the Emirati (citizen) adult population aged 20–70 years [3]. According to more recent estimates, the UAE has now been overtaken by other Gulf countries including Saudi Arabia, Bahrain, Kuwait and Qatar, however, the UAE still ranks 16^th^ in the world [4]. With a rapid improvement in socioeconomic status, the lifestyle of the Emirati population has changed considerably over the past 40 years, leading to less physical activity and altered eating habits [5].

Epidemiological studies in the UAE have predominantly focussed on the prevalence of diabetes and associated risk factors amongst the Emirati population. However, the UAE also has a large diverse multi-cultural and multi-national expatriate population accounting for approximately 85% of the total UAE population. In the past 40 years, the UAE has achieved remarkable economic growth, raising the prosperity of its citizens and creating opportunities for expatriates to migrate to live and work in the country. A significant proportion of immigrants include women, from Arab countries, South Asia and the Philippines who are typically employed as housemaids (female servants carrying out general domestic work in homes).

Previous studies have shown that exposure to environmental factors in economically developed countries may place immigrants at a higher risk of developing T2DM [6]. Upon arrival, immigrants are healthier than the native born population, however, their health status declines as the duration of their stay increases, as they adopt the cultural behaviors common in the host country, leading to changes in diet, physical activity level and environmental exposures [7, 8]. Various measures including language used or language proficiency of the host country have been used to capture this process of acculturation but the duration of stay in host country is one of the most common and reliable methods of measurement [9]. Currently, there is a lack of data concerning the prevalence of T2DM in migrant women in the UAE. As such, the main purpose of this study was to determine the prevalence of T2DM and examine the hypothesis that acculturation (measured by residency duration) is associated with an increased risk of T2DM among migrant women in the UAE.

## Materials and Methods

This study employed a cross-sectional design. Ethical approval was obtained from both the Al Ain Medical District Human Research Ethics Committee and the Abu Dhabi Ambulatory Healthcare Services Research Committee. Informed written consent was obtained from all the participants. All expatriate workers (including women) seeking employment in the UAE are required by law to undergo health and communicable disease screening (i.e. primarily pulmonary tuberculosis by chest X-ray and human immunodeficiency virus by serology) at a Government “visa screening center” before they can receive a residency permit. Expatriates are also required to undergo screening when they renew their visa which is usually every three years. The sampling frame in our study was a list of all the female expatriate workers (aged ≥18 years) who were enrolled for examination at the screening centre in Al Ain, Abu Dhabi, UAE. We used the formula for binomial distribution (n = Z^2^P (1-p)/d^2^) to estimate the sample size (n = 500) to identify the prevalence of diabetes in females. To account for refusals we increased the sample size and targeted 800 participants. We invited every third person on this list to participate in the study. Data collection for this study took place between 01 November 2012 and 31 May 2013. Exclusion criteria included female UAE citizens, female migrants from other countries (i.e. not Arab or South Asian countries or the Philippines), and females not providing consent. Arab included Egypt, Jordan, Palestine, Lebanon and Yemen while South Asian countries comprised.

India, Bangladesh, and Pakistan. We used an adapted version of the questionnaire used in the “STEPwise approach to Surveillance” (STEPS) developed by the World Health Organization (WHO) for the measurement and surveillance of non-communicable disease (NCD) risk factors in populations [10]. Due to the anticipated low literacy rates, the questionnaire was interviewer administered and interviews were conducted in Urdu, Bengali (India, Pakistan, Bangladesh), Tagalog (Philippines) or Arabic by a native research assistant from these countries who had received appropriate training. Data included demographic characteristics, lifestyle risk factors such as physical activity), family and personal disease history, occupation and monthly salary.

We ascertained T2DM as the self-report of a physician diagnosis of T2DMs and the use of a diabetic medication or a blood glycated hemoglobin level (HbA1c) level ≥ 6.5%. Pre-diabetes was defined as an HbA1c level of 5.7–6.4% [11]. Blood samples for HbA1c were tested at a local tertiary care hospital whose laboratory is College of American Pathologists (CAP) accredited. Our main exposure variable was length of residence in UAE as a marker for acculturation [9, 12]. We used two categories of years lived in the UAE (<10 years and ≥10 years). Body weight was measured to the nearest 0.1 kg using a calibrated electronic scale and a mounted stadiometer was used to measure height to the nearest 0.1 cm (SECA Hamburg, Germany). Weight and height measurements were completed with the participant wearing light clothing without shoes and standing motionless. Waist and hip circumference were measured using a flexible, non-stretch nylon tape measure (SECA Hamburg, Germany) while subjects were lightly clothed. Waist circumference was measured midway between the lower rib margin and the top of the iliac crest, in centimeters (cm) to the nearest 0.1 cm and hip circumference was measured at the point of maximal protrusion of the gluteal muscles to the nearest 0.1 cm. Body mass index (BMI) was calculated as body weight in kilograms divided by height in meters squared (kg/m^2^). The WHO cut-off points for BMI for adults were used to classify underweight (BMI <18.5 kg/m^2^), normal weight (BMI 18.5–24.9 kg/m^2^) overweight (BMI 25.0–29.9 kg/m^2^) and obese (BMI ≥ 30.0 kg/m^2^) [13]. We used a waist circumference (≥80.0 cm) to define central obesity [14]. Resting brachial blood pressure (BP) was measured using a calibrated automated device (Omron HEM-705cp) in sitting position using the right upper arm and an appropriate sized cuff after a period of five minutes rest. The average of two measures was used for analyses. Hypertension was defined as a mean systolic ≥ BP 140, a mean diastolic BP ≥90 or current hypertension treatment with prescription medication.

Information on physical activity was obtained using the International Physical Activity Questionnaire (IPAQ-short version) [15]; which measures the frequency (days per week), and duration (minutes per day) of moderate- and vigorous-intensity physical activity, in bouts of at least 10 minutes during the past seven-day period, globally in all contexts of daily life. The IPAQ also assesses the time spent walking (≥10 minutes duration) during the past 7 days and duration of walking within a given day in the last seven days was recorded to identify people who walked for at least 30 minutes each day. Two variables were computed from the IPAQ data: (i) proportion of participants that reported walking for ≥30 minutes per day; and (ii) proportion of participants reporting physical activity that would classify them as achieving the current public health recommendations (i.e. all healthy adults aged 18 to 65 years need moderate-intensity aerobic (endurance) physical activity for a minimum of 30 minutes on five days each week or vigorous-intensity aerobic physical activity for a minimum of 20 minutes on three days each week). Specifically, participants were dichotomised into either an *‘Active’* group (vigorous activity ≥ 3 d×wk^-1^ of ≥ 20 min×d^-1^, or ≥ 5 d×wk^-1^ of moderate-intensity activity and/or walking ≥ 30 min×d^-1^, or any combination of walking, moderate-, or vigorous-intensity activity, totalling ≥ 600 MET×min×wk^-1^) or an *‘Inactive’* group (not achieving the aforementioned level of activity) [16]. In the results section, the classification “moderate/vigorous physical activity” equates to the “active”group.

### Statistical Analysis

We entered the data using Microsoft Access before importation into Stata version 14.0 (StataCorp LP, College Station, TX) for analysis. Continuous variables were presented as means and standard deviations and categorical variables as counts and percentages. The crude prevalence of diabetes by nationality and duration of stay in the UAE were calculated as percentages with 95% confidence intervals (CI). Univariate logistic regression was used to calculate the un-adjusted odds ratio (and 95% CIs) of diabetes for a range of predictor variables including age, education, occupation, nationality, duration of UAE residence, family history of diabetes, physical activity, BMI category and waist circumference. Finally multiple logistic regression was used to produce odds ratios and 95% CIs, adjusted for those variables that were found to be significant in the simple logistic regression analysis.

## Results

Of the 800 eligible participants, 599 (75% participation rate) agreed to participate. A substantial proportion (24.4%) of eligible participants was prevented from taking part due to the demands of the visa screening process. Of the 599 who participated, 555 (92.6%) were from three geographical areas namely the Philippines (n = 292), Arab countries (n = 136) and South Asia (n = 127). The analysis that follows is confined this sub-set of 555 participants. The mean age was 34.1 ± 9.5 years and a majority (53.8%) came from urban areas within their home countries. With respect to accommodation arrangements, 43.5% lived with their family in the UAE, 35.6% lived with their sponsor (employer), 11.2% shared accommodation with non-relatives and 9.7% had single accommodation. Fifty seven percent were married with, on average, three children. Table 1 shows selected characteristics of the sub-set of study participants. A large proportion of the sample were classified as overweight (30.1%) or obese (16.8%) using BMI-derived estimates and nearly two thirds (65.8%) had central obesity based on waist-circumference derived estimates of abdominal adiposity. More than one quarter of the sample had a positive family history of T2DM (26.4%), a similar proportion self-reported walking for 30 minutes per day (24.4%) and nearly half of participants reported daily moderate-to-vigorous physical activity (46%).

**Table 1:** Characteristics of Female Migrants (n = 555) in Al Ain, United Arab Emirates, 2014.

Characteristic	n (%) / Mean ± SD
Age, years, Mean ± SD	34.1 ± 9.5
Age group	
18–29	210 (38.1)
30–34	125 (22.6)
≥35	217 (39.3)
Nationality	
Philippines	292 (52.6)
Arabs	136 (24.5)
South Asians	127 (22.9)
Education	
Primary or middle	39 (7.1)
Secondary	205 (37.5)
College or University	303 (55.4)
Monthly income, AED*	
<1000	136 (42.1)
1000–2000	83 (25.7)
>2000	104 (32.2)
Occupation	
Housemaid	214 (41.0)
Housewife	122 (23.4)
Professional	119 (22.8)
Administrator/Supervisor	44 (8.4)
Duration of stay in UAE, years	
<10 years	378 (79.1)
≥10 years	100 (20.9)
Anthropometric indices	
Height, cm	155.6 ± 6.7
Weight, cm	62.4 ± 15.0
Waist, cm	85.7 ± 13.3
Waist circumference category	
<80 cm	190 (34.2)
≥80 cm	365 (65.8)
Hip circumference, cm	98.0 ± 12.7
Waist-to-hip ratio	0.88 ± 0.1
Body mass index, kg/m	25.7 ± 5.8
Body Mass Index category	
Normal weight (<25 kg/m^2^)	295 (53.1)
Overweight (≥25–29.9 kg/m^2^)	167 (30.1)
Obese (≥30 kg/m^2^)	93 (16.8)
Blood Pressure, mmHg	
Systolic Blood Pressure	118.9 ± 18.6
Diastolic Blood Pressure	75.8 ± 12.7
Family history of diabetes	140 (26.4)
Physical Activity	
Walk for ≥30 minutes per day	92 (24.4)
Moderate/vigorous physical activity	227 (46.3)
Total number having HbA1c test	215 (38.7)
No diabetes (HbA1c < 5.7)	152 (70.7)
Pre-diabetes (HbA1c = 5.7–6.4%)	40 (18.6)
Diabetes (HbA1c ≥6.5%)	23 (10.7)
Physician diagnosed Diabetes	39 (7.3)

Numbers may not sum to 555 because of missing data.

SD: Standard Deviation.

Categorical variables were expressed as frequencies (percentages) and continuous variables as mean ± standard deviations.

* AED 1 = $0.27.

The following analysis is confined to the sub-set of 215 participants that had a blood test for HbA1c. Overall, 18.6% (95% CI: 13.9–24.4) had prediabetes and 10.7% (95% CI: 7.2–15.6) had T2DM. The prevalence of prediabetes was 8.5% (95% CI: 3.5–19.0) for Filipinos, 16.7% (95% CI: 10.2–25.9) for Arabs and 30.3% (95% CI: 20.3–42.5) for South Asians. Similarly the prevalence of T2DM was 1.7% (95% CI: 0.3–11.4) for Filipinos, 12.2% (95% CI: 6.9–20.9) for Arabs and 16.7% (95% CI: 9.4–27.8%). Table 2 summarises the number of cases (including measured and physician diagnosed cases), prevalence and odds ratios of T2DM for selected independent variables. Participants with T2DM were more likely to be 40 years or older, South Asian nationality, having a waist circumference 80 cm or more, positive family history of diabetes and involved in moderate level of physical activity. The prevalence of T2DM increased from 12.8% (95% CI: 8.4–19.1%) in those who had been resident in the UAE for less than 10 years to 40.7% (95% CI: 28.8–53.7%) in those resident for 10 or more years. This difference was statistically significant (p<0.001). Table 3 shows the results of the multiple logistic regression analysis. After adjustment, age group (AOR 3.48, 95% CI: 1.53–7.87), South Asian nationality (AOR 2.10, 95% CI: 0.94–4.70) and duration of UAE residence (AOR 2.74, 95% CI: 1.21–6.20) remained significant independent risk factors for T2DM.

**Table 2:** Cases of T2DM, prevalence and odds ratios* for selected independent variables.

Characteristic	All Diabetics (n = 44)‡		
	n (prevalence)	OR (95% CI)	P Value
Age group			
<40 years	19 (12.2)	Ref	
≥40 years	25 (42.4)	5.30 (2.62–10.73)	<0.001
Education			
Primary or middle	5 (27.8)	Ref	
Secondary	14 (15.1)	0.46 (0.14–1.50)	1.97
College or University	25 (24.8)	0.85 (0.27–2.63)	0.786
Occupation			
Housemaid/Professional	26 (18.1)	Ref	
Housewife	18 (25.3)	1.54 (0.78–3.05)	0.214
Nationality			
Arab/Filipino	24 (16.1)	Ref	
South Asians	20 (30.3)	2.26 (1.44–4.48)	0.019
Duration of stay in UAE			
<10 years	20 (12.8)	Ref	
≥10 years	24 (40.7)	4.66 (2.31–9.39)	<0.001
Waist circumference			
<80 cm	6 (10.0)	Ref	
≥80 cm	38 (24.5)	2.92 (1.17–7.33)	0.022
Body Mass Index Category			
Normal weight (<25 kg/m^2^)	17 (17.9)	Ref	
Overweight (≥25–29.9 kg/m^2^)	13 (17.3)	0.96 (0.43–2.13)	0.924
Obese (≥30 kg/m^2^)	14 (31.1)	2.07 (0.91–4.71)	0.082
Family history of diabetes			
No	25 (70.9)	Ref	
Yes	19 (53.7)	1.94 (0.98–3.86)	0.056
Moderate/vigorous physical activity			
No	10 (10.9)	Ref	
Yes	34 (27.6)	3.13 (1.46–6.74)	0.003

* Using simple logistic regression analysis.

‡ Comprises 23 cases with HbA1c ≥ 6.5% and 21 cases with self-report of a physician diagnosis of T2DM and the use of a diabetic medication.

**Table 3:** Adjusted odds ratios* of T2DM for selected independent variables.

Variables	All diabetics	
	AOR (95% CI)	P value
Age group		
<40 years	Ref.	
≥40 years	3.48 (1.53–7.87)	0.003
Nationality		
Philippines/Arab	Ref.	
South Asians	2.10 (0.94–4.70)	0.070
Duration of stay in UAE, years		
<10 years	Ref.	
≥10 years	2.74 (1.21–6.20)	0.016
Family history of diabetes		
No	Ref.	
Yes	1.21 (0.55–2.67)	0.639
Moderate/vigorous physical activity		
No	Ref.	
Yes	1.62 (0.68–3.89)	0.279
Waist		
< 80 cm	Ref	
≥ 80 cm	1.39 (0.50–3.86)	0.524

* Using multiple logistic regression.

AOR: Adjusted Odds Ratio (adjusted for age group, nationality, duration of stay in the UAE, family history of diabetes, moderate/vigorous physical activity and waist circumference), CI: Confidence Interval.

## Discussion

We have previously reported high levels of overweight, central obesity and hypertension amongst young South Asian male migrants in the UAE together with an association between length of residence (as a proxy for acculturation) and central obesity and diabetes [17]. In this study, in a sample of UAE migrant women, we have also found high levels of overweight (30.1%), obesity (16.8%) and central obesity (65.8%). The prevalence rate of T2DM was 10.7% with the highest rates in South Asian women. We also confirmed a significantly higher prevalence of T2DM associated with longer duration of residence. Specifically, after ten years, migrant women have three times the prevalence of T2DM compared with more recent arrivals to the UAE. After adjustment for income, education and occupation, our findings suggest that that age, South Asian nationality and length of residence of more than ten years are independent risk factors for T2DM amongst migrant women in the UAE. Our sample would appear to be representative of migrant women living and working in the UAE. Although exact demographic data are lacking in the UAE, it can be estimated that of the 9.2 million population (2015), two million are migrant women and of these 60% are from South Asia, Arab countries or the Philippines. In our sample, about half were from the Philippines, with the remainder from Arab and South Asian countries. There is an emerging body of evidence on the increasing prevalence of T2DM in South Asians, Arabs and Filipinos both in their home countries and abroad. For example, over the last two decades in South Asia there has been a significant increase in the prevalence of diabetes associated with urban residency, age, higher BMI, sedentary lifestyle and higher waist: hip ratio [18]. Previous research has shown that there is a high prevalence of T2DM amongst Filipinos and South Asians who have migrated to the United States [19, 20] and similarly these changes have been attributed to increasing levels of the traditional risk factors along with other factors such as the distribution of abdominal and visceral adiposity [21, 22]. Furthermore, analysis of data on Asian sub-groups from the United States National Health Interview Survey between 1997 and 2008 indicate an upward trend in BMI and T2DM prevalence [23]. Data comparing diabetes prevalence rates by country, nationality and ethnicity are published regularly but interpretation of these can be problematic because of differences in the case definitions that are used, heterogeneity of the study populations and variable time periods of data collection [24, 25]. Nevertheless, compared to women in their home countries, the prevalence of T2DM amongst women in our study was much higher for South Asian women (16.7% versus 6%) [26] and Arab women (12.2% versus 4–8%) [27] but lower for Filipino women (1.7% versus 6%) [28]. Also, the T2DM prevalence in our sample was lower than that reported amongst Emirati women (18%) [29].

Study findings revealed that the prevalence of T2DM was significantly higher among those who had been living in the UAE for ten years or more. Our study represents the first data available on the prevalence of obesity and diabetes in female Arab, South Asian and Filipino immigrants residing in the United Arab Emirates. However, similar findings have been reported for immigrants from these countries in North America and South America. For example, a study of immigrants in Canada found higher rates of diabetes among those from South Asia and those who had been living in Canada for 15 years or more compared to those living in Canada for 5–9 years [30]. We believe these findings are explained by *acculturation* which may be defined as the *adoption by immigrants of local customs and habits;* specifically, the adoption of western dietary habits which may result in adverse health effects such as obesity and T2DM [31]. Indeed, among participants in a multi-ethnic cohort study in the US, acculturation was found to be associated with higher diabetes prevalence in non-Mexican Hispanics due in part to BMI and diet [12]. Western dietary acculturation has been described in South Asian migrants in Canada including an increase in the consumption of convenience foods, sugar-sweetened beverages, red meat and meals taken outside the home [32]. Unhealthy dietary changes among Filipino immigrants in the US have also been reported including increased consumption of dairy products, meat, less starchy foods and snacks [33].

In previous studies, evidence for an effect of acculturation on weight gain and diabetes has been most convincing in men with less consistent results in women. Explanations for this difference include the possible effect of heritage cultural influences on body image, food choices and physical activity [34]. Alternatively, measurement and definition of acculturation may be the reason since in a longitudinal study of older Mexican immigrants in the United States, immigrant generation (having foreign-born parents) but not length of residence was associated with diabetes [35]. That said, in our study we found a significant association between acculturation (measured by length of residence) and diabetes in migrant women. Our findings provide further support for the worrying observation that the *Healthy Migrant Effect* or “immigrant health advantage” is temporary. On arrival, immigrants have a better health status than the native population but their health declines as their stay increases [36]. There are limited data on the behaviors that explain acculturation and its effects on BMI and diabetes prevalence. Among Filipino migrants, fructose intake from fruit-rich diets and loss of dietary diversity, have been offered as possible explanations [37]. Also, adoption of western dietary habits such as increased consumption of energy-dense convenience food may be less significant than the overconsumption of ethnic *festival foods*. These are food items high in carbohydrates, animal protein, sugar and fat that previously were consumed only few times a year on special occasions but which are now a routine part of the immigrant diet [38]. Although in our study 46% of subjects reported moderate or vigorous physical activity, it was less amongst Filipinos (24%) and previous studies have reported similar low levels [17, 39]. Amongst women migrants in the UAE there is clearly a need to address diet and lifestyle issues. However, little is currently known about the specific health behaviors of this important sub-section of the population or the barriers that prevent adoption of healthier behaviors. A study amongst Filipinos in Australia identified a range of possible barriers including personal resources, cultural influences and environmental factors [40]. Amongst South Asians, gender roles, body image, cultural factors and knowledge seem to influence behavior change [41]. Further research is required to investigate the dietary and behavioral factors that are contributing to the upward trend in overweight, obesity and T2DM in migrant women in the UAE. Such research is already planned amongst migrants in the Netherlands, a multi-ethnic high income country [42].

The strengths of this study include our random representative sample of women attending the mandatory health visa screening center in Al Ain, the second largest city in the emirate of Abu Dhabi in the UAE. Our response rate of 75% ensures that our sample is representative of migrant women in the UAE. We used an adapted version of the WHO STEPwise questionnaire for NCD risk factor surveillance, we used two anthropometric indices to classify obesity (BMI and waist circumference) and we objectively measured HBA1c (in a sub-sample of our study population). However, our findings must be interpreted in light of the acknowledged limitations of this study. Firstly, as with any cross sectional design, it is not possible to draw conclusions about the temporal or causal nature of the association between acculturation and the outcome variables. Secondly, we used years of residency to estimate acculturation rather than using a standardized acculturation measure. Currently, there are no validated instruments to measure acculturation amongst migrants in the Gulf region and the assessment of acculturation is culture -specific. Future researcher may wish to consider adapting tools that have been used in migrant populations in the UK and US. Alternatively, it may be appropriate to develop a new tool that is appropriate for our setting which might include dimensions such as dietary preferences, clothing style, use of own-culture media, language use and fluency, social connections, and cultural and religious beliefs.

In conclusion, there are high levels of overweight, obesity and central obesity amongst migrant women in the UAE and after ten years, migrant women have three times the prevalence of T2DM compared with more recent arrivals. It is likely that changes in diet and lifestyle account for these findings. Further research is needed to understand these changes but this should not delay the design and implementation of appropriate and feasible interventions aimed at the maintenance of a healthy body size and regular assessment of glucose control.
